# 3,9-Bis(2-chloro­phen­yl)-2,4,8,10-tetra­oxaspiro­[5.5]undeca­ne

**DOI:** 10.1107/S1600536810002400

**Published:** 2010-01-23

**Authors:** Xiao-Yong Wang, Jiang-Hua Shi, Min Zhang, Seik Weng Ng

**Affiliations:** a Department of Biology and Chemistry, Hunan University of Science and Engineering, Yongzhou Hunan 425100, People’s Republic of China; b Department of Chemistry, University of Malaya, 50603 Kuala Lumpur, Malaysia

## Abstract

The complete mol­ecule of the title compound, C_19_H_18_Cl_2_O_4_,  is generated by a crystallographic twofold axis that passes through the spiro C atom. The 1,3-dioxane ring adopts a chair conformation and the phenyl substituent occupies an equatorial site.

## Related literature

For the crystal structure of 3,9-diphenyl-2,4,8,10-tetra­oxaspiro­[5.5]undecane, see: Wang *et al.* (2006 ▶).
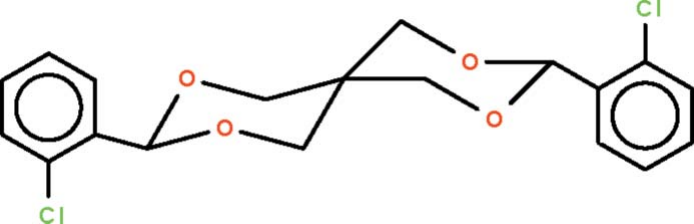


## Experimental

### 

#### Crystal data

C_19_H_18_Cl_2_O_4_
*M* *_r_* = 381.23
Monoclinic, 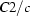

*a* = 10.7116 (5) Å
*b* = 9.4693 (5) Å
*c* = 17.7080 (9) Å
β = 106.745 (1)°
*V* = 1719.98 (15) Å^3^
*Z* = 4
Mo *K*α radiation
μ = 0.40 mm^−1^
*T* = 173 K
0.46 × 0.42 × 0.22 mm

#### Data collection

Bruker SMART APEX diffractometer
Absorption correction: multi-scan (*SADABS*; Sheldrick, 1996 ▶) *T*_min_ = 0.838, *T*_max_ = 0.917
6932 measured reflections
1883 independent reflections
1707 reflections with *I* > 2σ(*I*)
*R*_int_ = 0.015

#### Refinement

*R*[*F* ^2^ > 2σ(*F* ^2^)] = 0.032
*wR*(*F* ^2^) = 0.092
*S* = 1.00
1883 reflections
114 parameters
H-atom parameters constrained
Δρ_max_ = 0.30 e Å^−3^
Δρ_min_ = −0.25 e Å^−3^

### 

Data collection: *SMART* (Bruker, 2003 ▶); cell refinement: *SAINT* (Bruker, 2003 ▶); data reduction: *SAINT*; program(s) used to solve structure: *SHELXS97* (Sheldrick, 2008 ▶); program(s) used to refine structure: *SHELXL97* (Sheldrick, 2008 ▶); molecular graphics: *X-SEED* (Barbour, 2001 ▶); software used to prepare material for publication: *publCIF* (Westrip, 2010 ▶).

## Supplementary Material

Crystal structure: contains datablocks global, I. DOI: 10.1107/S1600536810002400/bt5174sup1.cif

Structure factors: contains datablocks I. DOI: 10.1107/S1600536810002400/bt5174Isup2.hkl

Additional supplementary materials:  crystallographic information; 3D view; checkCIF report

## Figures and Tables

**Fig. 1.**
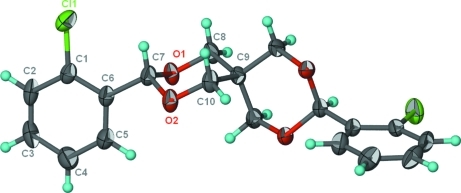
Anisotropic displacement ellipsoid plot of C19H18Cl2O4 at the 70% probability level; hydrogen atoms are shown as spheres of arbitrary radius.

## supplementary crystallographic information

### Experimental

Pentaerythritol (2 g, 0.014 mol), 2-chlorobenzaldehyde(4.6 g, 0.033 mol), toluene (12 ml) and a catalytic amount (0.2 g) of *p*-toluenesulfonic acid were heated for 4 hours. The mixture was cooled and then filtered. The organic phase was washed with water and 5% sodium bicarbonate (20 ml). The solvent was evaporated and the product recrystallized from ethyl acetate to afford colourless crystals (yield 70%); m.p. 418.5–419 K.

### Refinement

H-atoms were placed in calculated positions (C—H 0.95–0.99 Å) and were included in the refinement in the riding model approximation, with *U*(H) set to 1.2*U*(C).

### Crystal data

**Table:**

C_19_H_18_Cl_2_O_4_	*F*(000) = 792
*M**_r_* = 381.23	*D*_x_ = 1.472 Mg m^−^^3^
Monoclinic, *C*2/*c*	Mo *K*α radiation, λ = 0.71073 Å
Hall symbol: -C 2yc	Cell parameters from 5105 reflections
*a* = 10.7116 (5) Å	θ = 2.4–27.1°
*b* = 9.4693 (5) Å	µ = 0.40 mm^−^^1^
*c* = 17.7080 (9) Å	*T* = 173 K
β = 106.745 (1)°	Block, yellow
*V* = 1719.98 (15) Å^3^	0.46 × 0.42 × 0.22 mm
*Z* = 4	

### Data collection

**Table:**

Bruker SMART APEX diffractometer	1883 independent reflections
Radiation source: fine-focus sealed tube	1707 reflections with *I* > 2σ(*I*)
graphite	*R*_int_ = 0.015
ω scans	θ_max_ = 27.1°, θ_min_ = 2.9°
Absorption correction: multi-scan (*SADABS*; Sheldrick, 1996)	*h* = −13→13
*T*_min_ = 0.838, *T*_max_ = 0.917	*k* = −12→11
6932 measured reflections	*l* = −22→22

### Refinement

**Table:**

Refinement on *F*^2^	Primary atom site location: structure-invariant direct methods
Least-squares matrix: full	Secondary atom site location: difference Fourier map
*R*[*F*^2^ > 2σ(*F*^2^)] = 0.032	Hydrogen site location: inferred from neighbouring sites
*wR*(*F*^2^) = 0.092	H-atom parameters constrained
*S* = 1.00	*w* = 1/[σ^2^(*F*_o_^2^) + (0.0542*P*)^2^ + 1.3419*P*] where *P* = (*F*_o_^2^ + 2*F*_c_^2^)/3
1883 reflections	(Δ/σ)_max_ = 0.001
114 parameters	Δρ_max_ = 0.30 e Å^−^^3^
0 restraints	Δρ_min_ = −0.25 e Å^−^^3^

### Fractional atomic coordinates and isotropic or equivalent isotropic displacement parameters (Å^2^)

**Table:**

	*x*	*y*	*z*	*U*_iso_*/*U*_eq_	
Cl1	−0.05287 (3)	0.53900 (4)	0.14620 (2)	0.04156 (14)	
O1	0.30941 (9)	0.51739 (10)	0.13408 (5)	0.0283 (2)	
O2	0.31431 (8)	0.34130 (10)	0.22630 (5)	0.0298 (2)	
C1	−0.00068 (12)	0.40843 (14)	0.09327 (7)	0.0261 (3)	
C2	−0.09394 (12)	0.34711 (16)	0.03040 (8)	0.0329 (3)	
H2	−0.1815	0.3799	0.0160	0.040*	
C3	−0.05826 (13)	0.23835 (18)	−0.01081 (8)	0.0381 (3)	
H3	−0.1214	0.1960	−0.0539	0.046*	
C4	0.07000 (15)	0.19038 (18)	0.01047 (9)	0.0398 (3)	
H4	0.0941	0.1134	−0.0168	0.048*	
C5	0.16221 (13)	0.25578 (16)	0.07170 (8)	0.0342 (3)	
H5	0.2501	0.2242	0.0852	0.041*	
C6	0.12931 (12)	0.36636 (14)	0.11379 (7)	0.0259 (3)	
C7	0.23515 (12)	0.44177 (14)	0.17524 (8)	0.0265 (3)	
H7	0.1960	0.5084	0.2059	0.032*	
C8	0.40974 (12)	0.59806 (14)	0.18815 (8)	0.0303 (3)	
H8A	0.3692	0.6690	0.2148	0.036*	
H8B	0.4614	0.6491	0.1586	0.036*	
C9	0.5000	0.50311 (19)	0.2500	0.0240 (3)	
C10	0.41483 (12)	0.41154 (15)	0.28641 (7)	0.0291 (3)	
H10A	0.4699	0.3402	0.3216	0.035*	
H10B	0.3743	0.4715	0.3187	0.035*	

### Atomic displacement parameters (Å^2^)

**Table:**

	*U*^11^	*U*^22^	*U*^33^	*U*^12^	*U*^13^	*U*^23^
Cl1	0.0294 (2)	0.0446 (2)	0.0492 (2)	0.01101 (14)	0.00891 (16)	−0.00178 (15)
O1	0.0231 (4)	0.0307 (5)	0.0247 (4)	−0.0053 (4)	−0.0034 (4)	0.0058 (4)
O2	0.0220 (4)	0.0323 (5)	0.0290 (5)	−0.0030 (4)	−0.0024 (4)	0.0091 (4)
C1	0.0204 (6)	0.0298 (6)	0.0271 (6)	0.0012 (5)	0.0051 (5)	0.0078 (5)
C2	0.0182 (6)	0.0495 (8)	0.0282 (6)	−0.0041 (5)	0.0022 (5)	0.0102 (6)
C3	0.0283 (7)	0.0560 (9)	0.0275 (6)	−0.0157 (6)	0.0042 (5)	−0.0035 (6)
C4	0.0353 (7)	0.0459 (9)	0.0389 (8)	−0.0068 (6)	0.0120 (6)	−0.0098 (6)
C5	0.0223 (6)	0.0391 (7)	0.0390 (7)	0.0011 (5)	0.0055 (5)	−0.0028 (6)
C6	0.0194 (6)	0.0293 (6)	0.0262 (6)	−0.0012 (5)	0.0024 (5)	0.0052 (5)
C7	0.0191 (6)	0.0295 (6)	0.0278 (6)	0.0013 (5)	0.0016 (5)	0.0039 (5)
C8	0.0258 (6)	0.0259 (6)	0.0304 (6)	−0.0036 (5)	−0.0057 (5)	0.0046 (5)
C9	0.0208 (8)	0.0257 (8)	0.0214 (8)	0.000	−0.0004 (6)	0.000
C10	0.0224 (6)	0.0376 (7)	0.0232 (6)	−0.0006 (5)	0.0000 (5)	0.0051 (5)

### Geometric parameters (Å, °)

**Table:**

Cl1—C1	1.7384 (14)	C5—C6	1.388 (2)
O1—C7	1.4181 (15)	C5—H5	0.9500
O1—C8	1.4361 (15)	C6—C7	1.5062 (17)
O2—C7	1.4141 (15)	C7—H7	1.0000
O2—C10	1.4402 (15)	C8—C9	1.5272 (16)
C1—C2	1.3906 (18)	C8—H8A	0.9900
C1—C6	1.3921 (17)	C8—H8B	0.9900
C2—C3	1.378 (2)	C9—C8^i^	1.5272 (16)
C2—H2	0.9500	C9—C10	1.5293 (16)
C3—C4	1.392 (2)	C9—C10^i^	1.5293 (16)
C3—H3	0.9500	C10—H10A	0.9900
C4—C5	1.385 (2)	C10—H10B	0.9900
C4—H4	0.9500		
			
C7—O1—C8	110.41 (9)	O1—C7—C6	106.55 (10)
C7—O2—C10	110.16 (10)	O2—C7—H7	110.2
C2—C1—C6	121.55 (13)	O1—C7—H7	110.2
C2—C1—Cl1	117.40 (10)	C6—C7—H7	110.2
C6—C1—Cl1	121.04 (10)	O1—C8—C9	111.25 (10)
C3—C2—C1	119.41 (12)	O1—C8—H8A	109.4
C3—C2—H2	120.3	C9—C8—H8A	109.4
C1—C2—H2	120.3	O1—C8—H8B	109.4
C2—C3—C4	120.21 (13)	C9—C8—H8B	109.4
C2—C3—H3	119.9	H8A—C8—H8B	108.0
C4—C3—H3	119.9	C8^i^—C9—C8	107.86 (14)
C5—C4—C3	119.45 (14)	C8^i^—C9—C10	111.27 (7)
C5—C4—H4	120.3	C8—C9—C10	107.75 (7)
C3—C4—H4	120.3	C8^i^—C9—C10^i^	107.75 (7)
C6—C5—C4	121.57 (12)	C8—C9—C10^i^	111.27 (7)
C6—C5—H5	119.2	C10—C9—C10^i^	110.92 (16)
C4—C5—H5	119.2	O2—C10—C9	111.10 (9)
C5—C6—C1	117.72 (12)	O2—C10—H10A	109.4
C5—C6—C7	119.39 (11)	C9—C10—H10A	109.4
C1—C6—C7	122.75 (12)	O2—C10—H10B	109.4
O2—C7—O1	110.28 (9)	C9—C10—H10B	109.4
O2—C7—C6	109.32 (10)	H10A—C10—H10B	108.0
			
C6—C1—C2—C3	2.55 (19)	C8—O1—C7—C6	177.43 (10)
Cl1—C1—C2—C3	−176.83 (11)	C5—C6—C7—O2	−50.34 (15)
C1—C2—C3—C4	0.1 (2)	C1—C6—C7—O2	133.95 (12)
C2—C3—C4—C5	−2.1 (2)	C5—C6—C7—O1	68.83 (15)
C3—C4—C5—C6	1.5 (2)	C1—C6—C7—O1	−106.88 (13)
C4—C5—C6—C1	1.0 (2)	C7—O1—C8—C9	58.27 (13)
C4—C5—C6—C7	−174.90 (13)	O1—C8—C9—C8^i^	−171.63 (13)
C2—C1—C6—C5	−3.07 (19)	O1—C8—C9—C10	−51.41 (14)
Cl1—C1—C6—C5	176.29 (10)	O1—C8—C9—C10^i^	70.39 (14)
C2—C1—C6—C7	172.71 (12)	C7—O2—C10—C9	−58.74 (14)
Cl1—C1—C6—C7	−7.93 (17)	C8^i^—C9—C10—O2	169.66 (10)
C10—O2—C7—O1	64.19 (13)	C8—C9—C10—O2	51.61 (14)
C10—O2—C7—C6	−178.97 (10)	C10^i^—C9—C10—O2	−70.41 (9)
C8—O1—C7—O2	−64.03 (13)		

Symmetry codes: (i) −*x*+1, *y*, −*z*+1/2.

## References

[bb1] Barbour, L. J. (2001). *J. Supramol. Chem.* **1**, 189–191.

[bb2] Bruker (2003). *SAINT* and *SMART* Bruker AXS Inc., Madison, Wisconsin, USA.

[bb3] Sheldrick, G. M. (1996). *SADABS* University of Göttingen, Germany.

[bb4] Sheldrick, G. M. (2008). *Acta Cryst.* A**64**, 112–122. 10.1107/S0108767307043930 18156677

[bb5] Wang, J.-K., Yan, D.-Y., Liu, L.-J., Liu, S. & Wang, J.-T. (2006). *Acta Cryst.* E**62**, o3062–o3063.

[bb6] Westrip, S. P. (2010). *publCIF* In preparation.

